# Supplementary figures and images for: Mapping the Interactome of KRAS and Its G12C/D/V Mutants by Integrating TurboID Proximity Labeling with Quantitative Proteomics
Source: Biology (Basel). 2025 Apr 26;14(5):477. doi: 10.3390/biology14050477 (PMC12109396; doi:10.3390/biology14050477)

Related to Figure 2B

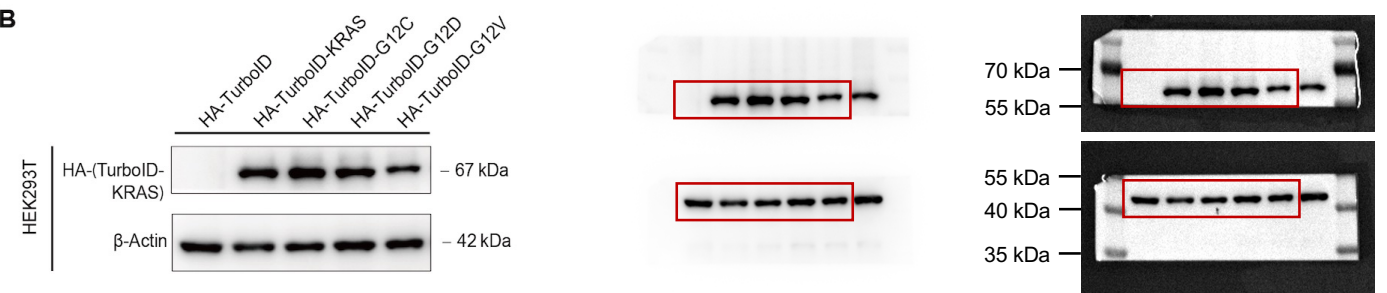

Related to Figure 2D

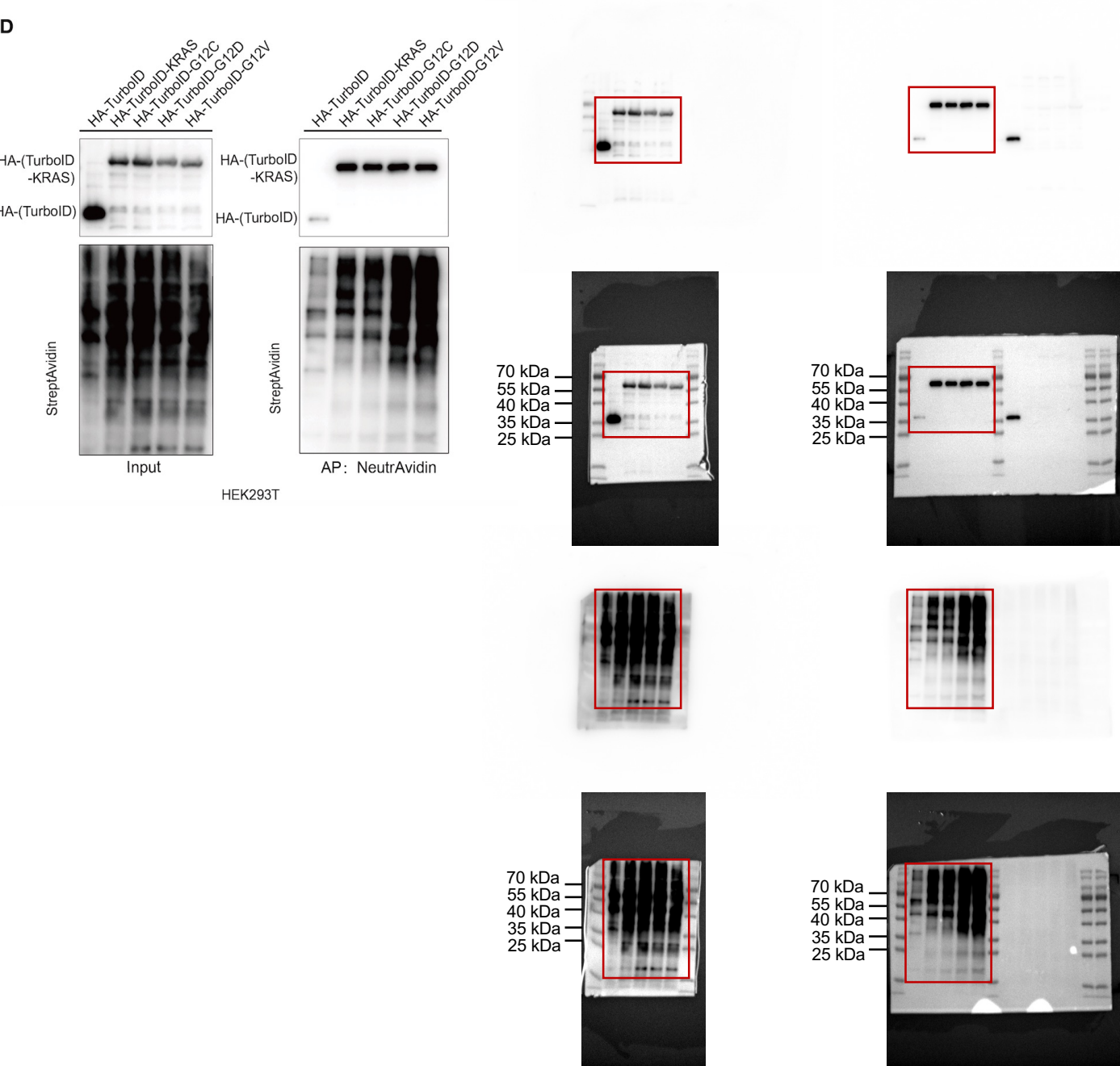

Related to Figure 4F

F

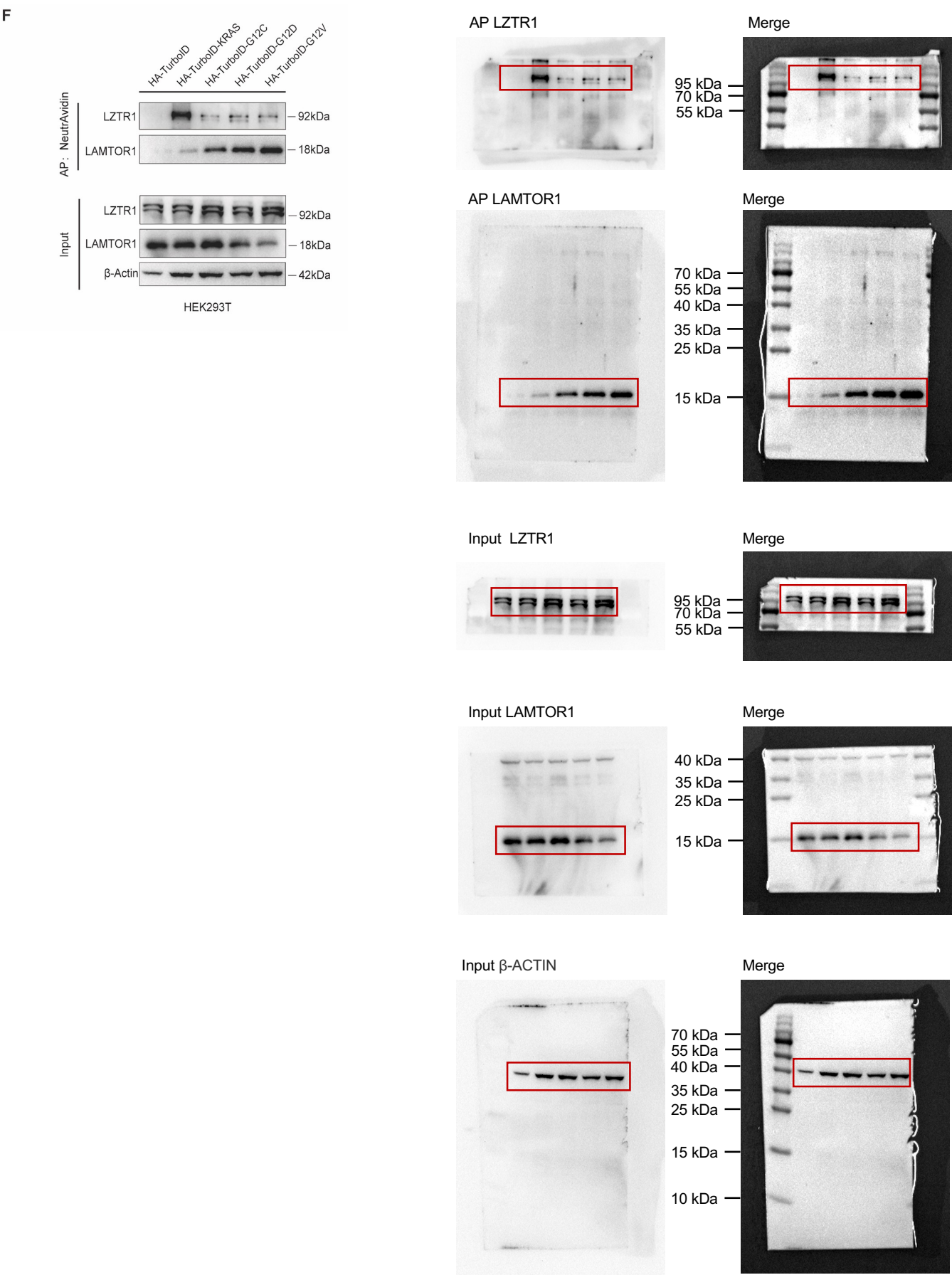

Supplement: Supplementary file 1 [file biology-14-00477-s001.zip › Figure S1.pdf]
